# Welfare Assessment in Shelter Dogs by Using Physiological and Immunological Parameters

**DOI:** 10.3390/ani9060340

**Published:** 2019-06-11

**Authors:** Cecilia Righi, Laura Menchetti, Riccardo Orlandi, Livia Moscati, Stefania Mancini, Silvana Diverio

**Affiliations:** 1 Istituto Zooprofilattico Sperimentale dell’Umbria e delle Marche “Togo Rosati”, via Salvemini 1, 06126 Perugia, Italy; cecilia983@tiscali.it (C.R.); l.moscati@izsum.it (L.M.); 2 Laboratory of Ethology and Animal Welfare (LEBA), Department of Veterinary Medicine, Perugia University, via San Costanzo 4, 06126 Perugia, Italy; laura.menchetti7@gmail.com; 3 Tyrus Clinica Veterinaria, Via Bartocci 1G, 05100 Terni, Italy; riccardo.orlandi83@hotmail.it; 4 Public Veterinary Services for Urban Hygiene and Prevention of Stray Dogs, USL Umbria 1, Municipal Rescue Dog Shelter, Strada per Brufa snc, Collestrada, 06148 Perugia, Italy; stefania.mancini@uslumbria1.it

**Keywords:** dog, shelter, health, immune system, β-endorphin, fecal cortisol, lysozyme

## Abstract

**Simple Summary:**

In “no-kill policy” countries, many dogs live in shelters. Several social, environmental, and management challenges can put the welfare of shelter dogs at risk. More knowledge is still needed on how to assess shelter dog welfare. This study aimed to evaluate the state of welfare of a group of dogs entering a shelter using physiological and immunological parameters by exploring the value of some biological indicators obtained by non-invasive methods. Considering that early welfare assessment could improve the management of subjects more prone to developing distress, measurements were taken at the time of admission and four weeks after the dogs entered the shelter. A multivariate statistical approach was used to comprehensively evaluate the relationship between the variables investigated. A reduction in the values of the measured physiological and immune parameters over time suggested an improvement in the dogs’ welfare after four weeks of being in the shelter compared to the initial capture and admission time. Findings also highlighted that some of the parameters investigated, such as neutrophils, lymphocytes, and fecal cortisol and lysozyme could be used for the welfare assessment of dogs entering a shelter.

**Abstract:**

This study aimed to evaluate the state of welfare of a group of dogs during the first month after entering the shelter by using different stress parameters. Blood and fecal samples were collected from a group of 71 dogs at the time of admission to the shelter. In 46 of these dogs, sampling was repeated after four weeks. Well-recognized welfare biomarkers, such as fecal cortisol and leukocytes, as well as some innovative parameters (β-endorphin and lysozyme) were determined. Uni- and multivariate statistical analyses were used to evaluate their interactions and changes over time. Neutrophils (*p* < 0.01), lysozyme (*p* < 0.05), and fecal cortisol (*p* < 0.05) decreased, while lymphocytes (*p* < 0.05) increased after four weeks compared to the first days of being in the shelter, suggesting an improvement in the dogs’ welfare over time. A principal component analysis extracted three bipolar components (PCs), explaining 75% of the variance and indicating negative associations between neutrophil and lymphocyte (PC1), lysozyme and β-endorphin (PC2), cortisol and lysozyme (PC3). The associations between these variables within each PC also confirmed the intricate relationships between the hypothalamic-pituitary-adrenal (HPA) axis and the immune system as well as the importance of a multiparametric approach in evaluating welfare.

## 1. Introduction

Nowadays, all over the world, a large number of dogs live in shelters. In several countries, including Italy, the euthanasia of sheltered dogs is prohibited. Despite the numerous positive ethical aspects, this “no-kill policy” has lengthened the duration of dogs staying in shelters, increasing the number of animals housed and public costs. Thus, the welfare of shelter dogs has become of considerable interest to professionals, researchers, and animal rescue organizations, as recently pointed out by the Association of Shelter Veterinarians [1,2]. Lack of social interaction, little exercise, minimal control over their environment, unpredictable noise levels, and caretaking routines can make living in a shelter stressful to dogs [3,4,5]. When an individual fails to cope and adapt to the stressful conditions, distress may occur, putting the welfare of the animal at risk [6]. Welfare assessment in shelter dogs is a topic still debated due to the complexity of the systems involved, the difficulty of defining indicators, and the adaptation skills of individual dogs [7].

The welfare of shelter dogs has been assessed in previous studies by measuring some physiological changes induced by long-term confinement [3,8]. The hypothalamic-pituitary-adrenal (HPA) axis plays a primary role both in acute and chronic stress responses. Stress response is, in fact, a part of daily life, but it is harmful when triggered too intensely or for too long a time [9,10]. Glucocorticoids, produced in the adrenal glands, are a key physiological tool in ecological studies and aid in the assessment of the health condition of populations as they cope with and respond to change [11]. Therefore, many scientists have measured cortisol as a biomarker in dogs by analyzing different matrices, such as blood, saliva, urine, fecal matter, and hair [5,12,13,14,15,16]. However, only a few studies have considered fecal cortisol to evaluate the welfare state in shelter dogs [17,18,19]. The use of feces as a matrix to measure adrenocortical activity gives some advantages compared to plasma. Fecal steroid metabolites provide a more representative measure over time because the pooling of metabolites during excretion reduces the episodic secretion of blood glucocorticoids. So the use of fecal steroids can mitigate the limitations of blood cortisol due to the circadian rhythm and pulsatile secretion [17,18,19]. Several authors have found an increase in urinary and plasma cortisol levels in the days immediately following admission of dogs to the shelter [5,8,20,21], while some have reported a reduction after a few days [20,21]. However, evaluation of cortisol changes is questionable, because a decrease in cortisol levels over time in shelter dogs could indicate habituation, but it could also be an incorrect response of the HPA-axis [7,20,22]. Besides, the relationship between cortisol level and chronic stress has not yet been defined univocally [7,20].

A multiparameter approach may help interpret cortisol levels and their changes regarding the welfare state of shelter dogs. First, cortisol measurement could be complemented by other HPA axis products. HPA axis activation also involves the secretion of corticotropin-releasing factor (CRH) and β-endorphin (BEP) [23]. BEP weakens the stress response, at least in part, by inhibiting secretion of CRH [24,25] and blocking stress-induced nociception [26,27,28]. Some authors have evaluated BEP in dogs [29,30], but their relationship with other stress indicators still needs to be clarified. 

Secondly, distress may lead to changes in the immune system of the animal, and it is well known that there are intriguing links between the HPA axis and the immune status [31,32,33]. Therefore, some hematological parameters, such as the relative number of circulating leukocytes, have been used as biomarkers of distress [34]. Recently other molecules like lysozyme, a component of innate immunity, have also been explored because they play a role in different degrees on the mechanisms of stress response [35,36]. In particular, lysozyme has been used as a psychological marker of stress in humans [32] and as a measure of distress in rats [34]. Stress causes a decrease in lysozyme levels, due to glucocorticoid secretion that inhibits the functioning of monocytes and macrophages. These latter are the principal sources of salivary lysozyme and an increase in the glucocorticoid level caused by stress can inhibit its production and secretion [36,37]. On the other hand, lysozyme has an important immune role with antimicrobial functions [38], but its role still needs to be fully elucidated.

Thus, the intricate interactions between the endocrine and immune responses to distress explain why animal welfare assessment can be extremely complicated. A multivariate statistical approach could be appropriate for studying a phenomenon where many variables are potentially involved and complex relationships exist between them. In particular, principal component analysis (PCA) is becoming increasingly popular among researchers to study complex and multidimensional phenomena such as in the health, biological, physical and social sciences, although it is still little used in veterinary medicine [39,40]. In the context of the present study, PCA could describe the structure of latent dimensions involved in the welfare of shelter dogs by including multiple parameters related to the HPA axis and the immune system. 

It is hypothesized that permanence in shelter induces changes both in the parameters related to the HPA axis and the immune system and that PCA could aid in understanding the relationships between these two systems and their interpretation with respect to welfare assessment. 

Thus, the aim of this study was to assess the welfare of dogs entering a shelter and after four weeks of residence by using some biomarkers of the HPA axis activity (fecal cortisol and BEP) and of the immune system activation (leukocytes, lymphocytes, neutrophils, monocytes, basophils, lysozyme), and to investigate the mutual relationship between the endocrine and immune system. Different biomarkers of stress, some well-recognized and some innovative, were simultaneously measured. Furthermore, those that are most useful, based on construct validity (i.e., logical associations between parameters) and predictive validity (attenuated responses over time) were identified. 

## 2. Materials and Methods

This study is part of a broader project, called “RandAgiamo^®^”, aimed at increasing the adoptability of adult shelter dogs in the Region of Umbria, Italy [2,41,42]. Moreover, this work was supported by the Italian Ministry of Health (“Evaluation of animal welfare in a confined animal population: a pilot study for the construction of a model for evaluating welfare in sheltered dogs”—Ricerca Corrente IZSUM RC122014).

### 2.1. Dog Shelters and Subjects

The study was conducted on a group of 71 stray dogs admitted to a shelter in Central Italy (the municipal shelter in Collestrada—Perugia, Umbria, Italy). Following capture, all dogs were immediately taken to the kennel and initially housed in single pens (average size of a single dog cage was 3 m × 1.5 m) as is the norm for newly captured dogs [43]. All pens had a roof for protection against adverse weather conditions and a kennel placed at the end. Ten days after capture, the dogs were moved to multiple (2–4 dogs) pens with both an open fenced and a covered area (size ranged from 15 to 40 m^2^) and a kennel for each dog. Dogs were fed once in the morning with commercial dry food, and fresh water was available ad libitum.

### 2.2. Experimental Protocol

The study was carried out for 14 months (March 2016–May 2017). The experimental protocol called for biological samples (blood and feces) to be collected from each subject two times: the first sampling was carried out within the first three days of admission to the shelter (Time 1 = T1), and the second sampling was four weeks after admission (acclimatization period; Time 2 = T2). 

### 2.3. Blood Samples

The collection of samples and manipulation of animals were carried out according to the shelter’s ethics code of practices. In particular, all blood samples were obtained according to the usual routine procedures of the kennel and after using them for the standard analyses they were also used for determining the other parameters investigated.

Blood samples (10 mL) were drawn by venous-puncture of the jugular vein of the dogs and collected in two different plastic vials, one of which containing EDTA. Vials were loaded in a rack, put in a transport box with cold packs and reached the laboratory within 6 h from collection. The EDTA vials were immediately processed for blood count (white and red blood cells, lymphocytes, neutrophils, monocytes, eosinophils, basophils) using an Exigo EOS bio 90 Boule Medical AB^®^ Analyser, while blood samples collected in the vials without an anticoagulant were centrifuged at 612 g for 10 min and serum samples were stored at −20 °C until analysis. β-endorphin concentrations were determined on the serum samples by a competitive inhibition enzyme immunoassay technique (CUSABIO^®)^ [44]. Once the reaction was stopped, the optical density of the plate (OD) was read using a Multiskan Go spectrophotometer (Thermo Fisher Scientific, Waltham, MA, USA), and Skanlt Software 4.1. The detection range was between 20 pg/mL and 4000 pg/m. In addition, the lysozyme concentration was measured by evaluating the lysis of Micrococcus lysodeikticus cells with the Lysozyme Detection Kit [45].

### 2.4. Fecal Samples

Wearing surgical gloves, fecal samples were manually collected from the rectal ampulla of each dog. They were then stored in individually labeled plastic bags and delivered to the laboratory and processed on the same day. The DetectX^®^ Cortisol Immunoassay (EIA) kit (Arbor Assays, Ann Arbor, MI, USA) was used to indirectly measure the cortisol response by determining the presence of its metabolites in the dogs’ faeces after organic phase extraction. The sensitivity limit was 17.3 pg/mL; the average intra-assay precision was 8.8%; the average inter-assay precision was 8.1%, and the cross-reactivity to corticosterone was 1.2% [46].

### 2.5. Statistical Analysis

Distributions within the demographic data were analyzed using Chi-square goodness-of-fit tests. 

The laboratory parameters (leukocytes, lymphocytes, neutrophils, monocytes, basophils, lysozyme, BEP, and fecal cortisol) were first subjected to univariate analysis. The assumptions were tested using the Kolmogorov-Smirnov test and diagnostic charts. Logarithmic transformation was used wherever appropriate (lysozyme and fecal cortisol values). Linear mixed models (LMM) were used to evaluate the differences between the first and second sampling times for each variable. These models included the dogs as subjects and the number of samplings (T1 and T2) as a repeated factor.

Therefore, cortisol, BEP, leukocytes, lymphocytes, neutrophils, monocytes, basophils, and lysozyme were subjected to multivariate analysis and, in particular, to principal component analysis (PCA). The parameters were included in the PCA after inspection of the correlation matrix to identify very low or very high correlations. The PCA determines if there are underlying variables in a complex set of correlations and associates the variables measuring the same dimension [39,47,48]. The Pearson correlation coefficient (r) was presented. Varimax rotation was used to increase the interpretability of principal components (PCs), and factor loading, i.e., correlation coefficients between the parameter and the PC after rotation is reported [47,48]. Parameters with cross loading issues (an item that has a high loading on two or more factors) were eliminated [42,48]. PCs having eigenvalues greater than 1 were retained and only factor loadings with an absolute value greater than 0.5 were interpreted and used to assign a PC label [48]. The corresponding PC scores were calculated using the regression method. The scores represent each individual’s placement on the factors identified by the PCA. These factor scores created three new variables (PC1–3) used for subsequent analyses [49]. Then, these PCs were analysed using the LMM [50] to evaluate the differences between T1 and T2.

All data were analysed using SPSS Statistics, version 23 (IBM, SPSS Inc., Chicago, IL, USA). Although *p* ≤ 0.05 was the accepted level of statistical significance, trends between *p* > 0.05 and *p* < 0.10 are also reported.

## 3. Results

### 3.1. Demographic Data of the Dogs

Out of the 71 dogs sampled at T1, only 46 were also available for the second sampling (T2) because 25 had been adopted or moved to other shelters before the end of the fourth week after entering the shelter. Among the sample population, no significant differences were found between males and females, while medium-sized dogs were the most represented (*p* < 0.001; Table S1). The mixed breed was the most represented (79.7%), while the most common coat colours were black and white, roan, fawn, and tricolour (*p* < 0.05). The presumed median age was 24 months (Table S1). 

### 3.2. Physiological and Immune Responses of the Dogs: Changes over Time (T1–T2)

**Figure 1:**
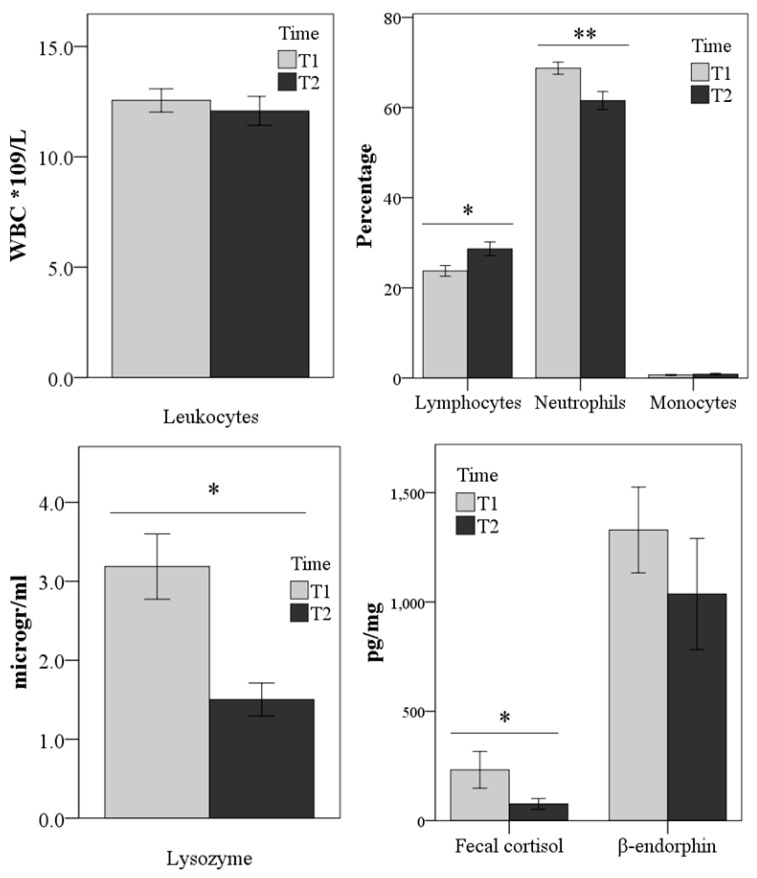
Differences in laboratory parameter values between the first (three days of shelter intake, Time 1 = T1) and the second sampling (after four weeks of acclimation in the shelter, Time 2=T2). ** *p* < 0.01, * *p* < 0.05 (univariate analysis). Basophils are not represented because they were constant.

Univariate analysis was first used to indicate the differences between the first and second sampling times for each parameter. The finding showed that neutrophils (*p* < 0.01), lysozyme (*p* < 0.05), and fecal cortisol (*p* < 0.05) decreased, while lymphocytes (*p* < 0.05) increased at T2 compared with T1 sampling (Figure 1). 

### 3.3. Principal Components Describing Laboratory Parameters

Cortisol, BEP, leukocytes, lymphocytes, neutrophils, monocytes, basophils, and lysozyme were next submitted to PCA, which describes their relationships and identifies latent dimensions. Variables to include in the analysis were preliminarily selected. The basophil variable was not included in the analyses because it was constant (0.0%). Moreover, the monocyte variable was eliminated because it was poorly correlated with the other variables (all correlation coefficients r ≤ 0.2; Table S2) and separated into a PC with low eigenvalues (1.002) and variance explained (<15%). In conclusion, the following variables were included in the PCA: leukocytes, lymphocytes, neutrophils, BEP, lysozyme, and fecal cortisol. 

The PCA revealed three components accounting for 73.5% of the total variance (Table 1). All the PCs were bipolar because they had a mixture of high positive and negative loadings. PC1 was named Neutrophil-Lymphocyte because neutrophil and lymphocyte items had high loadings with opposite signs. The other two PCs described the relationship between stress and the immune system and were called Lysozyme-β-Endorphin (PC2) and Cortisol-Leukocytes (PC3). In PC2, BEP and lysozyme had loadings of opposite sign, while in PC3 the fecal cortisol and leukocyte variables associated with the opposite directions. 

### 3.4. Principal Components over Time (T1–T2)

After calculating factor scores, the PCs were treated as new variables and analysed by LMM to check differences between the first and second sampling times. 

**Figure 2:**
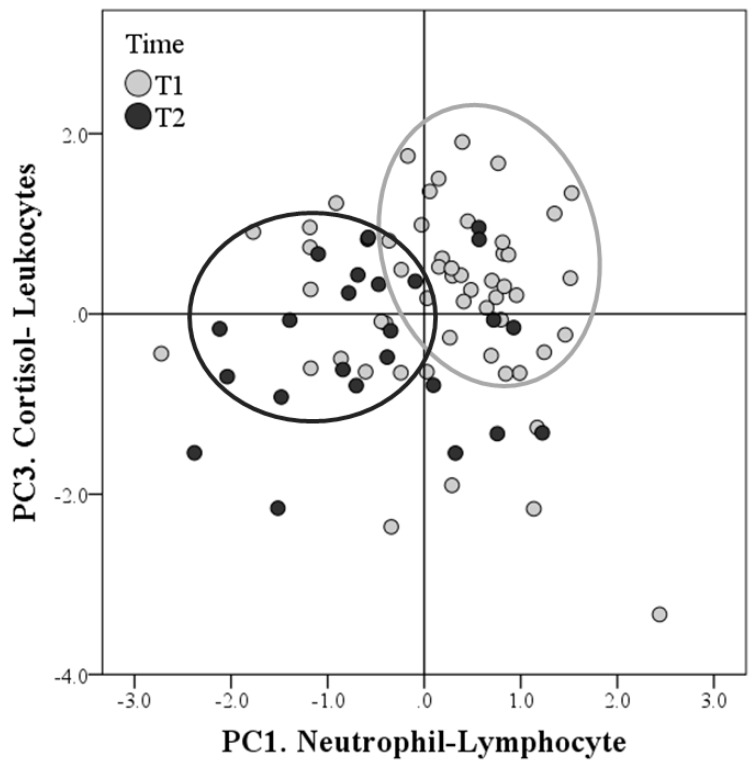
Factor maps of the principal component analysis (PCA). Distributions of the scores concerning the PC1. Neutrophil-Lymphocyte (x-axis) and PC3.Cortisol-Leukocytes (y-axis) extracted after PCA. The grey circles show that the scores of the first sampling (Time 1 = T1) are concentrated on the first quadrant in which both coordinates are positive. This suggests that many observations in T1 had positive scores for both PC1.Neutrophil-Lymphocyte and PC3.Cortisol-Leukocytes. On the contrary, many points of the second sampling (Time 2 = T2) have negative coordinates, especially as regards the x-axis. This suggests that many observations had negative scores in T2, especially for the PC1.Neutrophil-Lymphocyte.

**Figure 3:**
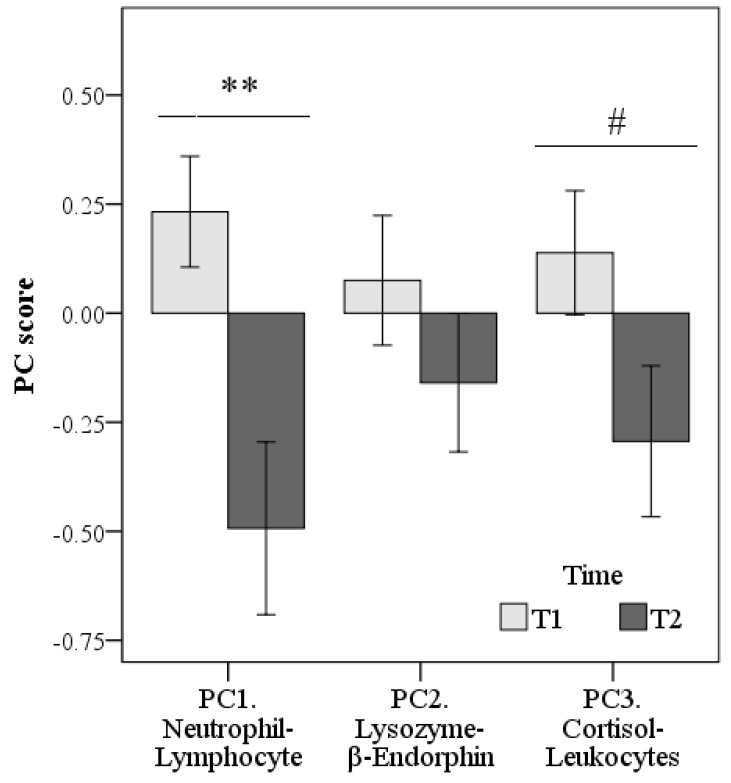
Differences in scores of PC1. Neutrophil-Lymphocyte, PC2.Lysozyme-β-Endorphin, and PC3.Cortisol-Leukocytes between the first (three days of shelter intake, Time 1 = T1) and the second sampling (after four weeks of acclimation in the shelter, Time 2 = T2). ** *p* < 0.01, # *p* < 0.1.

As highlighted by the score plot (Figure 2) and by the average PC scores on the bar graph (Figure 3), PC1.Neutrophil-Lymphocyte (*p* < 0.01) and PC3.Cortisol-Leukocytes (as trend, *p* = 0.071) decreased from the T1 to T2 sampling, while there were no significant differences for PC2.Lysozyme-β-Endorphin over time. 

## 4. Discussion

The Italian national framework law 281/1991 on prevention of stray dogs does not provide good practice standards for managing dogs in shelters [43]. Otherwise, at this regards, some dispositions are provided by the Italian regiolaws, which can differ by regions. This lack of uniformity in the regulatory frameworks to define minimum shelter requirements has hindered the development of a specific tool to assess shelter dogs’ welfare. The welfare of shelter dogs is of concern because many environmental factors may cause dogs to experience both acute and chronic stress [3,8,51,52]. Different approaches to assess the welfare of shelter dogs have been reported in the literature [5,12,14,15,53]. Recently, evaluation systems based on a multiple approach have been shown to be more reliable [53,54]. However, although attention to the welfare of shelter dogs is growing, more knowledge it is still needed due to the complexity of evaluating welfare quality. 

In the present study, the welfare status of a group of dogs was evaluated by using some biomarkers of the HPA axis activity and of the immune system activation in response to entering a shelter and after a four-week period of acclimatization. Furthermore, the mutual relationships between the endocrine and immune responses over time were also investigated. Finally, the use of some innovative parameters and statistical approaches to assess the welfare of shelter dogs is also introduced. 

Overall, our findings are in agreement with previous studies, which suggested that dogs can adapt to the kennel environment over time [8,16,21,55]. In particular, our findings show a decrease in the fecal cortisol levels after the acclimatization period of four weeks, compared with those recorded in the first three days in the shelter. During the initial physiological adaptive response, novel environment, manipulation, change in social structure, and different cages can represent sources of stress for dogs entering a shelter. Furthermore, it should be noted that the fecal cortisol measured in the present study indicates production during a certain timeframe [18]. Its level in dogs at the first sampling could also reflect the stressful conditions of street life experienced in the days preceding their admittance to the shelter. Anyway, after four weeks, the dog may have adapted to the new context, and the reduction of cortisol could indicate a decrease in stress. These findings support previous literature observations where circulating cortisol was found to be elevated during the first three days after capture, followed by a gradual decline [14,21]. However, some authors [15] pointed out that the decrease in cortisol is not a rule and long-term animal responses can be quite variable also depending on the individual and his/her past experience. The use of cortisol as an index of stress, especially in the long term, remains controversial. Indeed, cortisol concentrations may vary greatly and are subject to complex mechanisms also involving environmental, individual, and temporal variables [15,56,57,58]. Interpreting the low levels of cortisol found after four weeks in the shelter may, therefore, be difficult as they could indicate that the dog is no longer stressed or that the HPA axis of the dog is exhausted, as postulated by Selye in the third phase of the “general adaptation syndrome” [22]. In fact, very prolonged stress can lead to depletion of body energy resources and, eventually, to a decline of cortisol levels as the adrenals become “fatigued” and unable to continue to produce it. Thus, although glucocorticoid concentrations are the most practical and common measure for evaluating the functional activity of the HPA system, their levels in a single animal at any given point in time are affected by several other factors, not necessarily associated with stressful events [59]. Since blood collection, involving the need to handle the animal, could itself be stressful, the evaluation of fecal cortisol may have some advantage over plasma cortisol. Sampling the fecal matrix is not invasive, and fecal cortisol metabolite levels reflect an average level of circulating glucocorticoids during a time frame with a specific delay time [17], and thus, are not influenced by conditions related to that single point in time.

Interestingly, in the multivariate analysis, cortisol was negatively associated with leukocytes, confirming the close link between the HPA axis and the immune system. Moreover, although a trend, the principal component that describes this relationship, called PC3.Cortisol-Leukocytes, was lower after the acclimatization period in the shelter (four-weeks). 

Our findings showed significant changes over time also for neutrophils and lymphocytes, although always within the normal ranges. Neutrophils and lymphocytes decreased and increased, respectively, after four weeks of acclimatization in the shelter. Previous studies [7,60] reported similar results. Moreover, these two parameters were associated with opposite signs in the first component extracted by the PCA, explaining more than 30% of the data variance. The negative association between neutrophils and lymphocytes, as well as its implication in stress response, are not new topics in the literature. In fact, the Neutrophil/Lymphocyte (N/L) ratio has been used as a marker of stress in both humans and animals [31,34,61]. In agreement with the results of the univariate analysis, PC1.Neutrophil-Lymphocyte was higher in the first sampling, and decreased after the acclimatization period. This is in agreement with the literature [31,34,61,62,63,64] and confirms that numbers of neutrophils and lymphocytes are affected by stress in the opposite direction: neutrophils are elevated and lymphocytes depressed at admittance to the shelter, while this relationship is reversed after four weeks of residence. Therefore, the reduction of cortisol and the Neutrophil-Lymphocyte PC are in agreement, suggesting a decrease of stress after long term stay in the shelter. As mentioned above, it is hypothesized that dogs experience an acute and transitory stress response linked to capture, but do not perceive the subsequent sheltering period as a negative environment per se, as suggested by some authors [7,65]. It should be pointed out that during the second sampling, the animals were no longer isolated but in multiple pens. The different housing condition could be a critical fact from the experimental point of view, but it is the routine management practice of the shelter, and no changes were introduced so as to study the real conditions. However, the overall reduction in the stress response after the acclimatisation period may also confirm that multiple pens are beneficial for dog welfare [4,66,67]. Finally, the evaluation of multiple parameters should allow a better assessment of shelter dogs welfare even at the individual level, even considering the high individual variability [16].

Bearing this in mind, in a sample of dogs entering a shelter, the changes in lysozyme were also evaluated and, for the first time, BEP over time. Although the central action of BEP is not well studied, it is well known how during exposure to a stressor, the secretion of CRH and catecholamines stimulates, in turn, the secretion of the hypothalamic BEP, which inhibits the activity of the stress system [26]. Moreover, the role of lysozyme in immune and stress response has been documented although some of its functions remain unknown [34,38].

In the present study, the reduction of the lysozyme concentrations after four weeks from admission in the shelter is an interesting outcome. This change is not easy to be interpreted and it can be evaluated considering both the role of this enzyme and our dog population.

Lysozyme, secreted mainly by polymorphonuclear cells and leukocytes, has antibacterial properties interacting with microbial growth and metabolism [68,69]. It is reported how the concentration of this enzyme increases following inflammatory processes or bacterial infections, while it decreases in response to acute stress because of the immunosuppressive effects of glucocorticoid secretion that inhibits the functioning of monocytes and macrophages [36,38]. It is interesting to note that this inverse association between glucocorticoids and lysozyme was not confirmed in the present study. In fact, we found the highest concentrations of this enzyme at the time of shelter admission, when the cortisol was also higher, while we recorded a decrease of both parameters after four weeks from admission in the shelter. In our opinion, these results could be related to the specific study population of this work represented by stray animals. The poor environmental conditions in which these stray dogs were forced to live before entering the shelter, could have continuously stimulated the immune system in the face of adverse stimuli, leading to a significant increase in lysozyme concentration. In this perspective, a transient increase in stress linked only to the time of capture and the initial period inside the shelter, may not be sufficient in our population to cause a marked drop in the immunity expressed by lysozyme. A further value that supports this hypothesis is related to the mean level of lysozyme (3.19 mg/L) found in the serum of the dog population during the first sampling, similar to the salivary lysozyme concentration (3.17 mg/L) reported by Iacopetti [69] although in saliva it is usually 9 times more concentrated than in serum. 

From the above, the reduction of lysozyme found after the acclimatization period could reflect an adaptive physiological response expression of an improvement in the general welfare. Finally, the lack of correlation between cortisol and lysozyme, as well as with all other parameters, could be also due to the fact that they were measured in different matrices, feces, and blood, reflecting the physiological response in a temporally disconnected way. Overall, these findings support that lysozyme plays a role in stress response, but partially disconfirm results of previous studies [36,38]. Therefore, further investigation in dogs is needed. 

On the contrary, the results of the BEP, both with the univariate and PCA, were inconsistent since no significant differences were found over time. These inconsistent results can be explained in two ways: (i) the entry into the shelter can generate variable responses of β-endorphin on dogs that were already experiencing a previous stressful condition, and (ii) the high variability of β-endorphin affects the statistical analysis and does not allow significant differences to be revealed between the first and second samplings. 

However, the inverse relationship between BEP and lysozyme that emerged with the multivariate analysis is, to our knowledge, unpublished, and merits reflection. This link could reflect the attempt to achieve, over time, an adaptive physiological response against stressors at different levels of the stress system. We hypothesize how the central role of BEP, stimulated after CRH and catecholamine secretions [70], plays a later role in modulating the stress response with respect to lysozyme. In particular, lysozyme should act in the first moments of the stress response, enhancing the immune system, while the main role of BEP should be related to its capacity to inhibit the stress system at the pituitary level when triggered for too long [71,72,73]. In conclusion, it is possible that BEP and lysozyme are working together at different times, trying to prevent the adaptive response from becoming maladaptive.

Some limitations of the current study should be pointed out. A more extended period of observations after admission to the shelter and a larger sample size could give a more accurate analysis of quite variable parameters such as BEP concentrations. Further insights may be gained by analyzing the relationship among the physiological and immunological parameters investigated in this study with the behavioral assessments made on the same sample of shelter dogs [67].

## 5. Conclusions

Multiple and innovative parameters (physiological, and immunological) are proposed to evaluate the welfare of dogs entering a shelter as well as the use of an original statistical approach. Among all parameters investigated, neutrophils, lymphocytes, fecal cortisol, and lysozyme changed consistently over time, suggesting that they could be used for the evaluation of the welfare of shelter dogs. Moreover, changes in these parameters seem to reflect adaptation to the shelter environment and living conditions. This study also highlighted the important and complex role of lysozyme as a possible biomarker of distress in dogs, while inconsistent results were obtained with BEP. However, further evaluation of both lysozyme and BEP is needed. 

The multivariate analysis confirmed the intricate but close link between the HPA axis and the immune system. Indeed, cortisol and β-endorphin were negatively associated with leukocytes and lysozyme, respectively. This statistical approach seems to offer interesting potential in the study of a multidimensional phenomenon such as the state of animal welfare.

## Figures and Tables

**Table 1:** Factor loadings for the laboratory parameters included in the principal component analysis.

Item	Component		
	PC1. Neutrophil-Lymphocyte	PC2. Lysozyme-β-Endorphin	PC3. Cortisol-Leukocytes
Lymphocytes %	−0.934	0.098	−0.030
Neutrophils %	0.907	−0.014	0.062
β-Endorphin (Pg/Ml)	0.088	−0.826	0.202
Lysozyme (Microgr/Ml)	−0.007	0.783	0.272
Fecal Cortisol (Pg/Mg)	0.280	0.022	0.782
Leukocytes (WBC *109/L)	0.455	−0.027	−0.619
% Variance explained	33.93	21.60	17.93
Cumulative % variance explained	73.47		

Loadings of ≥ |0.50| are bolded.

## Supplementary Materials

The following are available online at https://www.mdpi.com/2076-2615/9/6/340/s1, Table S1: Demographic characteristics of the dog; Table S2. Matrix of the Pearson correlation coefficients.
