# Supplementary material for: Welfare Assessment in Shelter Dogs by Using Physiological and Immunological Parameters
Source: Animals (Basel). 2019 Jun 11;9(6):340. doi: 10.3390/ani9060340 (PMC6616394; doi:10.3390/ani9060340)
Supplement: Supplementary file 1 [file animals-09-00340-s001.pdf]

SUPPLEMENTARY MATERIAL

Table S1. Demographic characteristics of the dog

	Number	Percentage[#]	Chi square	P value
**Gender**				
**Female**	32	46.4%	0.36	0.547
**Male**	37	53.6%		
**Age** (months; median, first and third quartile)	24 (12, 36)		-	-
**Size**				
**Small**	17	24.6%	48.38	<0.001
**Medium**	*49*	*71.0%*		
**Large**	3	4.3%		
**Breed**				
**Mixed**	*55*	*79.7%*	286.60	<0.001
**Group 1-** Sheepdogs and Cattledogs*	1	1.4%		
**Group 2-** Pinscher and Schnauzer - Molossoid and Swiss Mountain	1	1.4%		
**Group 3 –** Terrier	1	1.4%		
**Group 6-** Scent hounds and related breeds	5	7.2%		
**Group 7-** Pointing Dogs	3	4.3%		
**Group 8 -** Retrievers - Flushing Dogs - Water Dogs	2	2.9%		
**Pitt bull**	1	1.4%		
**Coat**				
**Black and white**	*11*	*28.9%*	17.42	0.026
**Red and white**	3	7.9%		
**Fawn**	*5*	*13.2%*		
**Red**	3	7.9%		
**Gold - cream**	2	5.3%		
**Black**	2	5.3%		
**White**	0	0.0%		
**Liver**	1	2.6%		
**Tricolour**	*5*	*13.2%*		
**Grey**	*6*	*15.8%*		

The prevailing category on the total number of participants is indicated in Italics (Chi-Square Goodness of Fit Tests). Breed types were categorised according to Federation Cynologique Internationale (Federation Cynologique international n.d.).

Table S2. Matrix of the Pearson correlation coefficients. *P<0.05, **P<0.01 (2-tailed).

	**Fecal Cortisol (Pg/Mg)**[a]	**β-endorphin (pg/mg)**	**Leukocytes (WBC *109/L)**	**Lymphocytes (%)**	**Neutrophils (%)**	**Monocytes (%)**
**β-Endorphin (Pg/Ml)**	0.077					
**Leukocytes (WBC *109/L)**	-0.120	-0.045				
**Lymphocytes (%)**	-0.185	-0.054	-0.238*			
**Neutrophils (%)**	0.173	-0.002	0.180	-0.782**		
**Monocytes (%)**	0.150	-0.078	0.054	-0.151	0.060	
**Basophils (%)** [b]	-	-	-	-	-	-
**Lysozyme (Microgr/Ml)**[a]	0.099	-0.204*	-0.101	0.061	-0.037	-0.126

a After logarithmic transformation.

b Cannot be computed because at least one of the variables is constant.
